# Using a Region-Based Convolutional Neural Network (R-CNN) for Potato Segmentation in a Sorting Process

**DOI:** 10.3390/foods14071131

**Published:** 2025-03-25

**Authors:** Jaka Verk, Jernej Hernavs, Simon Klančnik

**Affiliations:** Laboratory for Machining Processes, Faculty of Mechanical Engineering, University of Maribor, Koroška Cesta 46, 2000 Maribor, Slovenia; jaka.verk@student.um.si (J.V.); jernej.hernavs@um.si (J.H.)

**Keywords:** image segmentation, potato sorting, neural network, mask RCNN, object detection, production process, machine learning, AI

## Abstract

This study focuses on the segmentation part in the development of a potato-sorting system that utilizes camera input for the segmentation and classification of potatoes. The key challenge addressed is the need for efficient segmentation to allow the sorter to handle a higher volume of potatoes simultaneously. To achieve this, the study employs a region-based convolutional neural network (R-CNN) approach for the segmentation task, while trying to achieve more precise segmentation than with classic CNN-based object detectors. Specifically, Mask R-CNN is implemented and evaluated based on its performance with different parameters in order to achieve the best segmentation results. The implementation and methodologies used are thoroughly detailed in this work. The findings reveal that Mask R-CNN models can be utilized in the production process of potato sorting and can improve the process.

## 1. Introduction

### 1.1. Sorting Process in Industrial Environment

The sorting process is a critical component of the food industry, as it ensures that consumers receive produce of high quality. By removing defective or contaminated items, effective sorting extends shelf life, enhances food safety, and prevents disease. Additionally, it improves consumer acceptability, contributing to overall product quality and industry standards [1]. The sorting process generally necessitates the evaluation of produce quality by workers, a task that is often labor intensive, time consuming, and cost prohibitive, particularly for large quantities. To address these challenges, there has been a growing adoption of automated sorting systems based on vision technology.

Automated sorting is a key factor in enhancing productivity, reducing production costs, and ensuring the quality of products in the food and agriculture industry. Conventional sorting methods frequently depend on manual visual inspection or rudimentary computer techniques, such as threshold segmentation and classical computer-vision methodologies. Nevertheless, these techniques are often laborious, vulnerable to variations in lighting conditions, and incapable of the precise instance segmentation of individual objects in intricate images [2]. These limitations have prompted the adoption of more advanced approaches, particularly those leveraging deep learning.

The utilization of deep neural networks, particularly convolutional neural networks (CNNs), is witnessing a marked rise in the agricultural sector as a potent instrument for classification. This assertion is substantiated by numerous studies that have demonstrated the efficacy of CNN-based methodologies in a myriad of agricultural applications, encompassing soil quality assessment and the high-precision classification of crops such as potatoes [3,4]. CNN architectures also employ object detection, a capability that facilitates a multitude of applications in the agricultural domain. These include crop-density detection, disease detection, blemish detection, and crop detection [3,5,6]. Current research is focused on the combination of CNN methods with other machinery, such as robotic manipulators and drones, which allows for the automation and simplification of processes in the food industry, including crop harvesting and sorting [3,7,8].

Among the various CNN-based approaches, Mask R-CNN has gained significant attention for its ability to perform precise instance segmentation. CNN architectures for object detection including R-CNN, TridentNet and YOLO (You only look once) enable the detection of multiple objects in an image, essential for automated sorting systems, with the output of only bounding boxes, while Mask R-CNN distinguishes itself by generating highly accurate segmentation masks, thereby ensuring precise instance segmentation [9,10]. Despite advancements in the field of neural networks for image segmentation, there exist many challenges regarding the practical usage of Mask R-CNN in the real production environment. The key problems include:

- Performance and speed of execution: Mask R-CNN models are often computationally demanding, which affects image processing time and, consequently, the throughput of the sorting line.
- Model optimization for industrial usage: The selection of software and hardware can significantly impact the efficiency of the algorithm in real-time applications.
- Comparison of implementations: Mask R-CNN can be implemented in various software environments, with MATLAB offering rapid prototyping, while Python provides a more flexible workflow and optimization of the model with the help of libraries, like TensorFlow and PyTorch.

Research has demonstrated the efficacy of Mask R-CNN in agricultural contexts. Studies have underscored its superiority in applications necessitating exact instance segmentation, such as the accurate segmentation of on-tree fruit and the estimation of their size despite occlusions, surpassing the capabilities of the Vision Transformer model [11]. Other studies have demonstrated its usability in conjunction with drones for the identification of invasive weeds in agricultural fields and the isolation of soil regions from complex backgrounds, enhancing preprocessing for soil-type identification. These studies suggest a potential enhancement of crop management when employed in conjunction with aerial drones [12,13]. The studies have also demonstrated the applicability of Mask R-CNN for the detection of crop growth and the estimation of livestock weight. A study was conducted to create an enhanced Mask R-CNN model for estimation of lettuce’s growth status, allowing for optimal harvest timing. In another study, a modified model was designed with the main goal of weight prediction of live pigs. The study incorporated depth images, enhancing the accuracy of the prediction process, a feature lacking in the study of lettuce growth [14,15].

The advantages of Mask R-CNN extend beyond agricultural usage, as evidenced by its successful application in various domains, including medical imaging and automated food processing, where precise segmentation is critical. For instance, a study developed a pick-and-place robot that successfully sorted small food packets despite occlusions [16]. Another study developed a fairly accurate calorie-estimation model using Mask R-CNN, which helped detect food on a plate and calculate the number of calories on the plate. A comparative analysis was conducted with Faster R-CNN, and Mask R-CNN exhibited superior performance in this regard. This efficacy was further substantiated in a separate study where a neural network was trained to detect COVID-19 disease in computed tomography (CT) images [17,18].

The sorting process in the study entailed the sequential operation of an object detection model and a classification model. To optimize the classification outcomes with the second model, Mask R-CNN was selected for object detection due to its capacity for instance segmentation, which involves the removal of the background from each segmented potato. To achieve the best balance of precision and processing time, the study analyzes the implementation of Mask R-CNN in two environments (MATLAB and Python) and evaluates which training parameters are the most appropriate for automated potato sorting.

### 1.2. Research Aims

The study focuses on the development of an accurate potato instance segmentation to be used in an automated industrial sorting process. Segmentation in the study is made with Mask R-CNN architecture, which is evaluated in the study based on results under various parameters. The utilization of Mask R-CNN in this study is predicated on its superior capacity for precise instance segmentation, a property that has the potential to ensure superior classification. Classification is anticipated to perform more effectively, as it operates on a small image patch, thereby improving accuracy by ignoring the background while simultaneously reducing computational requirements. The study analyzes the efficiency of Mask R-CNN architecture in potato segmentation and presents optimal parameter settings for usage in the real potato-sorting process. This study hypothesizes that, when optimized for hardware and software selection, Mask R-CNN can achieve highly accurate segmentation through segmentation masks while enhancing computational efficiency for industrial potato sorting.

### 1.3. Contribution

This paper contributes to the field of industrial agricultural automation by focusing on optimizing the use of Mask R-CNN for segmentation in an automated sorting process. While previous research has explored Mask R-CNN applications in various domains such as food classification [17] and COVID-19 detection [18], this study uniquely applies it to real-world potato sorting, addressing key challenges such as segmentation accuracy, execution speed, and industrial feasibility. Unlike previous work on potato classification using CNNs [4] and blemish detection using AdaBoost [5], this study goes beyond mere object detection to achieve precise instance segmentation, which is critical for accurately classifying potatoes of varying quality in real time. In addition, it systematically compares program architecture implementations and training variables, demonstrating that software choice and parameter tuning significantly affect segmentation accuracy and processing speed. Furthermore, compared to general agricultural AI applications [3], this work directly addresses industrial deployment issues such as computational efficiency and robustness under variable lighting conditions. The results, particularly the superior performance of a model (mAP = 0.878), highlight the importance of software and variables optimization over brute-force training (i.e., increasing epochs).

## 2. Materials and Methods

### 2.1. Segmentation

Segmentation is a process in image processing that identifies objects and determines their classification, defining what is displayed in an image and where specific objects are located. There exist numerous types of segmentation, including semantic and instance segmentation. In semantic segmentation, each pixel is assigned a specific class, and once each pixel is classified, adjacent pixels are grouped to form objects of a particular classification. The key distinction between semantic and instance segmentation is that instance segmentation differentiates between individual objects within the same class. Moreover, in instances of segmentation, the necessity of individual pixel classification is not universally applicable, as objects can be segmented as whole entities, contingent upon the type of neural network employed for instance classification [10].

Segmentation enables the identification of objects in an image, thereby allowing for subsequent decision-making based on the extracted information. In manufacturing processes where multiple objects appear in a single image, instance segmentation is predominantly utilized. This approach allows for the detection and individual processing of multiple objects of the same classification within a single frame. Due to this capability, instance segmentation was chosen for the development of the sorting system. Consequently, the camera’s field of view could capture multiple potatoes simultaneously, thereby accelerating the sorting process and increasing the number of potatoes sorted per hour.

### 2.2. Mask R-CNN

**Figure 1:**
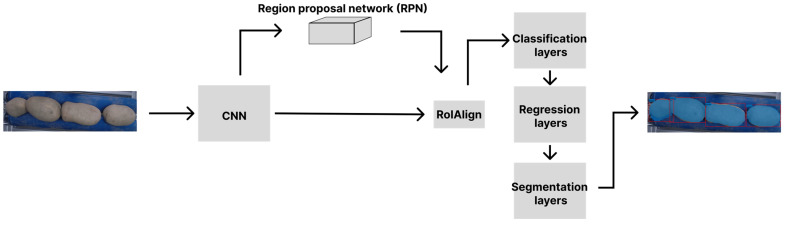
Mask R-CNN architecture. The input and output images are displayed, with the results of the instance segmentation results overlaid onto the output image.

Mask R-CNN, developed by the Facebook AI Research (FAIR) team (Meta Platforms, Menlo Park, CA, USA), is one of the various R-CNN neural network methods by which instance segmentation is carried out. Instance segmentation performed with neural network architecture R-CNN and its derivatives (i.e., Fast R-CNN and Faster R-CNN) identifies object instances within an image and returns their bounding boxes and labels [10]. The key distinction between these methods and Mask R-CNN, a method built upon the Faster R-CNN architecture, is its capacity to not only generate bounding boxes and labels, but also to produce a segmentation mask for each object, enabling Mask R-CNN to provide precise pixel-level information regarding object regions, a functionality not attainable with other R-CNN methods (Figure 1) [10].

For this key reason, Mask R-CNN was chosen for the segmentation process. This enabled the forwarding of cropped images of each potato instance to a secondary neural network dedicated to classification. The pixel values corresponding to the potato instance were preserved, while all other pixels were set to 0, thereby simplifying classification by eliminating irrelevant background information.

### 2.3. Hardware and Software Utilized

Images of potatoes for training and evaluation were taken with Basler acA2500-14gc (Basler AG, Ahrensburg, Germany). The entire training and evaluation process was conducted on an Asus ROG Flow X16 (2022) GV601RM laptop (ASUSTeK Computer Inc., Taipei, Taiwan) equipped with an NVIDIA GeForce RTX 3060 laptop GPU with a TDP of 125W (NVIDIA Corporation, Santa Clara, CA, USA). The process was executed using MATLAB 23.2 (MathWorks, Natick, MA, USA) and the Python programming language, version 3.10.14 (Python Software Foundation, Beaverton, OR, USA). Python was implemented within the Anaconda environment (Anaconda, Inc., Austin, TX, USA), which included the following essential libraries: OpenCV 4.7.0 (OpenCV.org, Kizhevsk, Russia), pip 23.3.1 (Python Software Foundation, Beaverton, OR, USA), and PyTorch 2.2.2+cu118 (Meta Platforms, Inc., Menlo Park, CA, USA). Additionally, the NVIDIA CUDA 11.8 architecture (NVIDIA Corporation, Santa Clara, CA, USA) was utilized to optimize GPU-based computations.

### 2.4. Research Process

The research commenced with the acquisition of a dataset of images of potatoes for training, followed by the preprocessing and organization of images and data. Subsequently, a training dataset was prepared through manual annotation of potato instances. Following the processing of all data, the neural network training phase was initiated. The final step involved the evaluation of all trained neural networks and a comparative analysis of the obtained results.

### 2.5. Image Preparation

A series of images were captured using a camera while the potatoes were positioned on the rollers of the sorting machine. The potatoes could have been placed on any surface; however, it was determined that placement on rollers would be optimal for the sorting system. This setup was expected to enhance both the neural network’s performance and segmentation accuracy. By rotating the rollers, the potatoes were turned, allowing images to be taken from multiple angles. In a practical application, rotation of the potatoes would also be necessary, as decay might be present only on one side.

Potential improvements in the classification of potato decay were explored by capturing infrared-spectrum images using a thermal camera. Decayed sections of the potatoes appeared colder than the healthy areas.

The images containing multiple potatoes were categorized into three distinct groups: rotten potatoes, feed potatoes, and high-quality potatoes. Rotten potatoes exhibited clear signs of decay, feed potatoes were either bruised or green (unsuitable for retail sale), and high-quality potatoes met the standards for commercial distribution. The potatoes were categorized by an expert that evaluated the quality of each potato.

**Figure 2:**
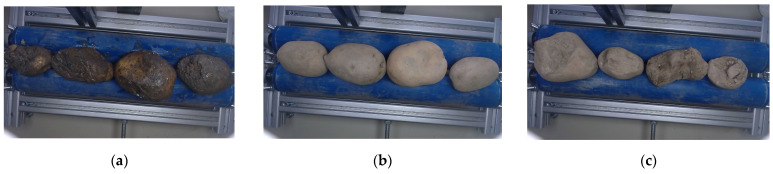
Example images of potatoes used for training: (**a**) rotten potatoes; (**b**) high-quality potatoes; (**c**) feed potatoes.

A total of 696 images were collected, with 105 images of rotten potatoes, 375 images of feed potatoes, and 216 images of high-quality potatoes (Figure 2). The distribution of images utilized for training and evaluation of the neural networks is presented in Table 1. Of the total, 521 images were selected for training, comprising all potato categories, with a predominant focus on feed potatoes (70.5%) and rotten potatoes (19.7%). This emphasis was necessary because, in some cases, the difference between mud-covered rollers and the potatoes was minimal, potentially affecting classification accuracy.

The selected images were imported into the MATLAB programming environment, where they were processed and prepared for neural network training using the built-in Image Labeler application (Computer Vision Toolbox 23.2). During the preparation process, a class of polynomial annotations named “Potato” was created. Each potato in the images was outlined with a polygonal annotation, ensuring that the number of labeled objects corresponded to the number of potatoes in the image. The annotations were made as precisely as possible; however, due to the polygonal nature of the labels, perfect accuracy was not attainable. The total number of labeled objects created across all images was 2028, with the number of potatoes per image ranging from three to six, and an average of nearly four potatoes per image.

Following the completion of the manual segmentation of the selected images, the annotation files in MATLAB were saved as “labelingProject.prj”, which contained the progress of the annotations, and “gTruth.mat”, which stored a dictionary named “Ground Truth”. This dictionary included all data regarding the location of the processed images, the number of object classes present in each image, and the coordinates of the polygons outlining these objects.

### 2.6. Preparation of the Neural Network in MATLAB

Using the gTruth.mat file, data on the manually labeled images, including information on the polygons of actual objects in the images, were imported into the neural network training program. Based on these polygons, the boundary coordinates of the bounding box for each object were calculated. In addition to calculating the bounding box for each polygon, logical images were created for each polygon, with the same size as the images containing the potatoes. The logical image was structured such that every pixel within the polygon was marked with a value of 1, while pixels outside the polygon were marked with a value of 0.

Once all the necessary data were obtained using various functions in MATLAB, the parameterization of the Mask R-CNN neural network training settings began. During the training of different neural networks, only the number of epochs, over which the network was trained, was modified. Therefore, the training settings common to all models in MATLAB are presented in Table 2.

In the future, all neural networks will be numbered sequentially and labeled as NNn (Neural Network n), where “n” represents the neural network number in order.

Neural Network 1 (NN1) was trained on 10 epochs of images with dimensions 1080 × 1920. One epoch represents one complete cycle over the entire dataset intended for training. In this case, the dataset consisted of all images designated for neural network training. Consequently, if NN1 underwent 10 epochs of training, it completed 10 cycles over the entirety of the dataset [19]. Neural Network 2 (NN2) was trained on 500 epochs of images with dimensions 480 × 270. The third neural network (NN3) was trained on 500 epochs of images with dimensions 1280 × 720. The fourth neural network (NN4) was trained on 1000 epochs of images with dimensions 480 × 270.

The dimensions of the images were modified during the training of the neural networks for several reasons. The objective was to attain equal or superior segmentation quality at the minimum possible image size, as this affects numerous segmentation parameters. By reducing the image size, the aim was to shorten the training time of the neural network, as large neural networks can make the training process very time consuming. The required image size for segmentation also impacts the selection and acquisition of the camera integrated into the sorter, as the larger the required image size, the more expensive the camera. The image size also influences the storage space occupied by each image, which, in addition to occupying space, affects the speed of image transfer and processing during segmentation. All these parameters ultimately influence the costs and speed of sorter operation.

After setting up the neural networks, the Mask R-CNN neural network architecture was imported, and training commenced based on the dictionary created specifically for training. The dictionary contained polygons, polygon masks, bounding boxes, and image locations.

### 2.7. Preparation of the Neural Network in Python

The neural network was implemented also in Python, as this programming language facilitates more seamless integration with other languages and exhibits superior speed compared to MATLAB. Given that the images had previously been processed and labeled with regions containing potatoes in the MATLAB environment, the decision to process the data in Python was more straightforward. However, it should be noted that Python does not natively support reading .mat files. To address this limitation, the gTruth.mat file was converted into a gTruth.json file using the MATLAB function exportGroundTruthToJSON. A salient benefit of this format is its capacity to ensure the legibility of JSON files by humans [20].

The PyTorch library was utilized to construct the neural network, a process that necessitated the adaptation of the data format. Logical mask images were generated from polygon information with pre-prepared functions, and the images were stored in a dictionary, facilitating the efficient processing of individual images.

Prior to training the neural network, the images underwent random transformations, including posterization, grayscale conversion, contrast adjustment, brightness modification, and horizontal flipping. Each transformation was applied with a probability ranging from 10% to 50%. These transformations were implemented to ensure the neural network’s functionality under diverse lighting conditions within the sorting system, given that the original images were captured under uniform lighting conditions, since a study comparing YOLOv8 and Mask R-CNN proved that Mask R-CNN detected objects worse under different lightning conditions [21].

Once the images were collected and properly processed with transformations, the training of the neural network (NN5) followed, using a dataset of images with dimensions 512 × 512 for 40 epochs.

The different learning parameters of the trained neural networks are shown in Table 3.

### 2.8. Preparation of Images for the Evaluation of Neural Networks

A total of 164 images of potatoes on rollers were included in the evaluation set, none of which had been used for training the neural network, thereby ensuring the most realistic results for assessing the network’s performance. This was due to the fact that the network was exposed to new images that it might encounter in practical applications. To optimize the evaluation process, the images were cropped to a dimension of 1920 × 580, as the rollers were located only within this section of the full image, originally sized 1920 × 1080, captured by the sorting system’s camera. Cropping the images also had practical value in the sorting program, as only the areas where potatoes could appear were analyzed, reducing the likelihood of errors.

The images designated for evaluation were processed manually to ensure that all objects belonging to the potato class were labeled. These images were then transferred into a ground-truth file, which contained both the images and the corresponding object data. The file format was identical to the one used for training the neural network, except that it contained different images and objects. The images were subsequently prepared for evaluation in Python, as well as in MATLAB, using the methods presented in the following section.

### 2.9. Methods for Evaluating Instance Segmentation

The performance of neural networks can be evaluated in several ways. In all methods, a comparison is made between the actual polygon areas of objects from the ground-truth file and the polygon areas of objects obtained from the neural network. Actual objects refer to the instances of objects that appear in each image (e.g., four potato objects), while segmented objects represent those detected by the neural network. The number of actual objects and segmented objects in an image may differ.

**Figure 3:**
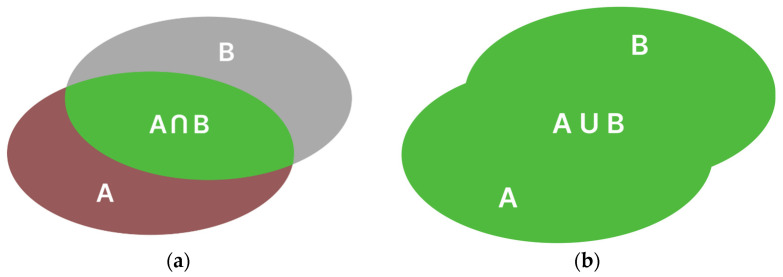
Symbolic representation: (**a**) intersection of sets; (**b**) union of sets.

The first metric for evaluating the performance of a neural network is IoU (Intersection over Union). IoU represents the ratio of the intersection area of the actual object polygon and the segmented object polygon to the area of the union of these polygons (Equation (1)) (Figure 3). The IoU parameter is a dimensionless value ranging from 0 to 1, where 1 indicates perfect overlap between the masks, while 0 indicates no overlap between the masks [22,23,24].

(1) $$IoU=\frac{A\;\cap \;B}{A\;\cup B}$$

This method is highly advantageous for comparing masks of individual objects; nevertheless, it is not optimal for assessing the overall suitability of the neural network. It is possible for too few objects to be detected, yet these objects may possess a high IoU value. Consequently, the average IoU value is calculated, despite the fact that not all objects were accurately identified.

To address this challenge, the mAP parameter is frequently employed. This parameter is founded on three concepts: precision (P), recall (R), and average precision (AP). Prior to delving into the properties of these parameters, it is essential to elucidate the methodologies employed to assess the segmentation of individual objects.

**Figure 4:**
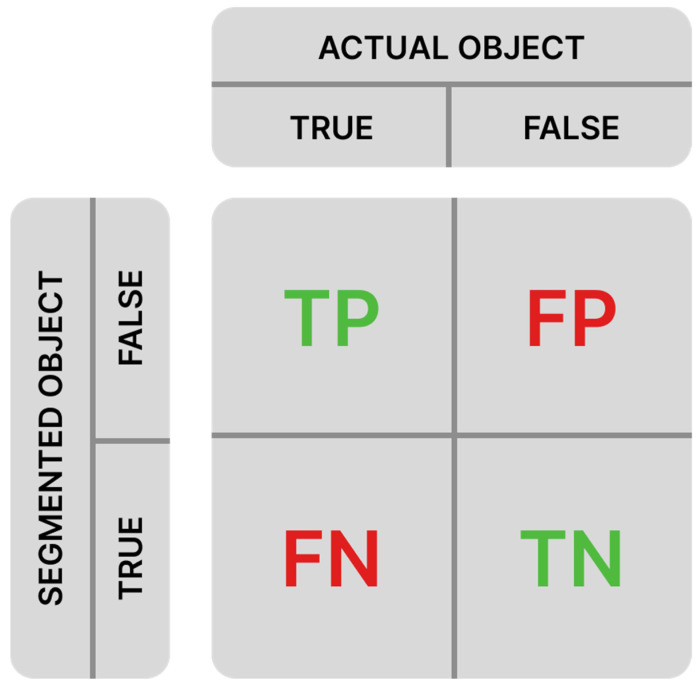
Confusion matrix.

Each segmented object is classified into three categories determined by the comparison of its IoU value and the established overlap threshold [10]: TP (true positive), FP (false positive), and FN (false negative). TN (true negative), which represents background that was not segmented as an object, is also known. A confusion matrix (Figure 4) is used for the classification of the segmented object, which graphically illustrates into which category the segmented object falls.

Precision (P) is defined as the ratio of the number of TP classifications to the total number of segmented objects (Equation (2)). Recall (R) is calculated as the ratio of the number of TP classifications to the total number of actual objects (Equation (3)) [25].

(2) $$P=\frac{TP}{TP+FP}$$

(3) $$R=\frac{TP}{TP+FN}$$

The evaluation of neural networks is based on a comparison of precision and recall, which results in a graph of the precision–recall relationship. Since recall frequently does not range between the limits of 0 and 1, including the boundary values, n recall values were defined, evenly distributed between 0 and 1. In this way, precision values were interpolated based on recall, which allowed for the creation of a graph with n connected points. This produced a continuous function that illustrates the previously mentioned relationship. To quantify this relationship, the average precision (AP) parameter was calculated, representing the area under the continuous precision–recall curve, enabling a facile evaluation of the neural network’s performance based on the AP value. AP can range between 0 and 1, where 1 indicates perfect neural network performance at a given overlap threshold. The AP value is calculated as the average of the maximum interpolated precision values based on n evenly distributed recall values from 0 to 1 [22,24].

(4) $${AP}_{n}=\frac{1}{n}\;\sum_{R\;\in \;[0,1]}P_{interp}\;(R)$$

To obtain the mean average precision (mAP) for all specified precision thresholds, the average of all AP values is easily calculated. In the equation used to calculate mAP, the variable N represents the number of calculated AP parameters, which is equal to the number of overlap thresholds used (Equation (5)) [22,24].

(5) $$mAP=\frac{1}{N}\;\sum_{i=1}^{N}AP_{i}$$

The subsequent parameter that facilitates the evaluation of the neural network using recall and accuracy is the *F*1 score, which provides a straightforward perspective on the relationship between recall and accuracy of the neural network, akin to the AP value. The closer the *F*1 score value is to 1, the superior the performance. The method selected for calculating the *F*1 score necessitates the average value of all recalls and accuracies [25,26].

(6) $$F1=\frac{2}{P^{-1}\cdot R^{-1}}=\frac{2\cdot P\cdot R}{P+R}$$

Given that the equation is performed by multiplying the average values of recall and accuracy, it is impossible for the *F*1 score to reach a value of 1, as the recall values are distributed between 0 and 1.

In order to compare the performance of the neural network, the values of AP and *F*1 were compared at different overlap threshold values. Therefore, it was necessary to create multiple graphs of the relationship between accuracy and recall based on different overlap threshold values.

## 3. Results

### 3.1. Evaluation Results

For each evaluation of the neural network, the confidence threshold at which the best results were obtained was ascertained (see Table 4). All neural networks were evaluated at the following overlap threshold values: 0.1, 0.3, 0.5, 0.7, 0.72, 0.74, 0.76, 0.78, 0.80, 0.82, 0.84, 0.86, 0.88, 0.90, 0.92, 0.94, 0.96, and 0.98, with the exception of NN3, which was evaluated solely at the overlap thresholds of 0.1, 0.3, 0.5, 0.75, and 0.90, as will be explained subsequently. This signifies that, in Equation (5), for all neural networks:

N = 18,(7)

except for NN3, where

N = 5,(8)

Table 4 presents the results of the average accuracy and average F1 score of the neural networks. In these results, an increase in the mAP value can be observed depending on the number of epochs used for training each neural network, with deviations appearing in NN3 and NN5 [27].

**Figure 5:**
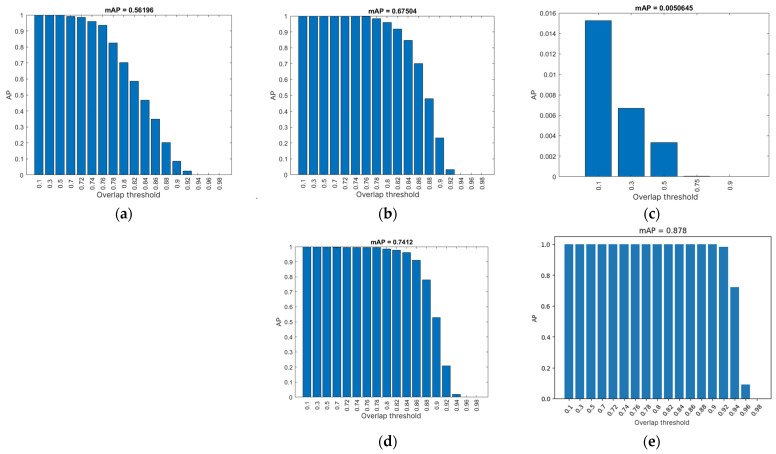
AP value based on overlap threshold: (**a**) NN1; (**b**) NN2; (**c**) NN3; (**d**) NN4; (**e**) NN5.

**Figure 6:**
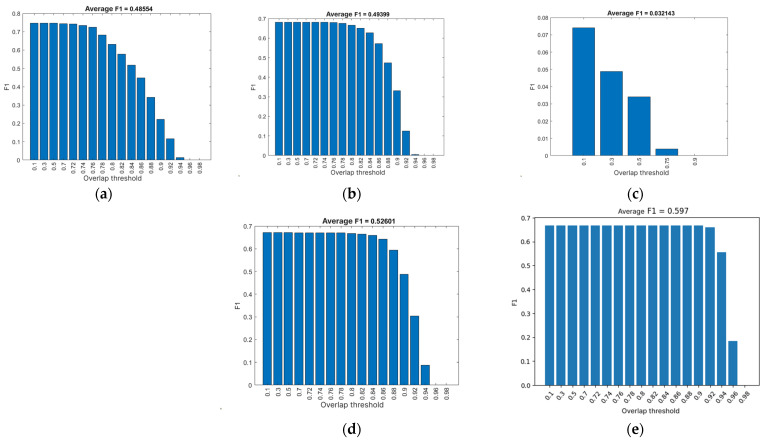
F1 score based on overlap threshold: (**a**) NN1; (**b**) NN2; (**c**) NN3; (**d**) NN4; (**e**) NN5.

**Figure 7:**
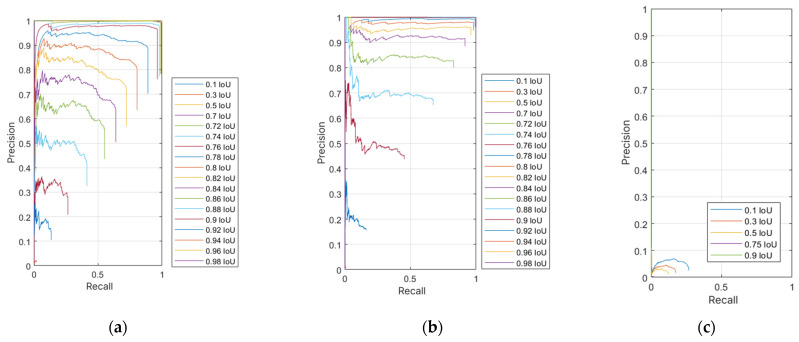

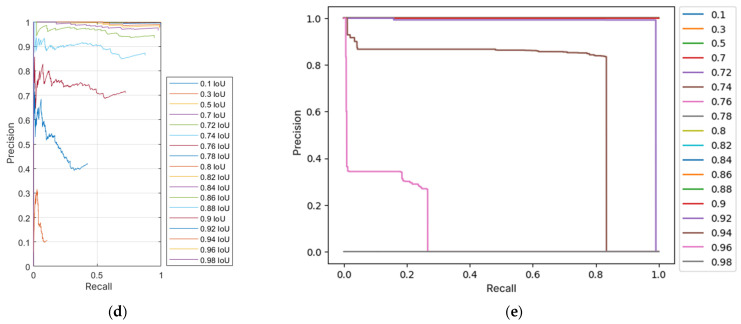
Precision–recall dependency: (**a**) NN1; (**b**) NN2; (**c**) NN3; (**d**) NN4; (**e**) NN5.

**Figure 8:**
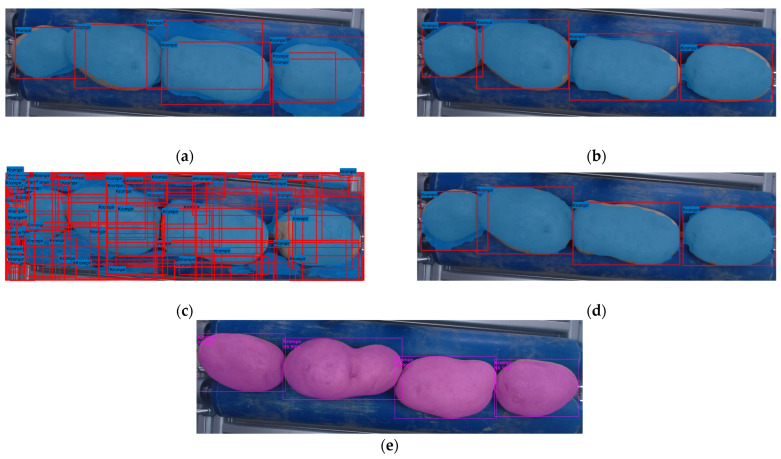
Visual representation of segmentation. The images display the bounding boxes and masks of instance segmentation results overlaid on the input images.: (**a**) NN1; (**b**) NN2; (**c**) NN3; (**d**) NN4; (**e**) NN5.

NN3 exhibited a marked deviation from the established results, attributable to an unidentified error that occurred during the training of the neural network. A comprehensive evaluation of the neural network revealed that it consistently yielded the poorest outcomes, as evidenced by the data presented in Table 4 and further elucidated in Figure 5c, Figure 6c and Figure 7c. Given the conspicuously unsatisfactory outcomes, it was determined that further evaluation of the neural network at an overlap threshold greater than 0.75 would be futile, as the AP value approached zero even at this threshold. The neural network in question exhibited the lowest AP value among all neural networks, falling short of two percent. The aberrant behavior of the neural network is evident in the segmentation performed by this network, as depicted in Figure 8. The erroneous segmentation exhibited by NN3 renders it unfit for practical application in an industrial setting, underscoring the potential for untoward errors during the training of neural networks, which can compromise their efficacy.

Neural network NN5 yielded the most optimal results in the evaluation process. This particular neural network demonstrated the highest mAP value and the F1 score. The AP (Figure 6d) exhibits a decline only at an overlap threshold of 0.92, reaching a nadir at the threshold of 0.98 ultimately reaching zero. When compared to other neural networks, NN5′s AP demonstrates its most precipitous decline at the latest stage. This outcome suggests that the neural network generates precise results, as it accurately detects all potatoes at an overlap threshold of 0.9, a capability not exhibited by the other networks. NN5 also attains the maximum F1 score, as illustrated in Figure 7d, where the graph closely resembles the one in Figure 6d. However, it is important to note that the F1 score cannot attain a value of 1, as the average recall and precision are never simultaneously equal to 1. In the precision–recall ratio graph (Figure 8d), it is evident that recall only begins to decline at overlap thresholds of 0.92 and beyond. This graph can be logically connected to the one in Figure 6d, as the declines are visible at the same overlap threshold values.

This finding underscores the notion that an increase in the number of epochs does not guarantee optimal performance. Instead, a strategic change in the programming language can yield substantial enhancements in neural network performance based on the different architecture offered by the programming environment. A comparison of NN5 with the second-best neural network, NN4, which was trained for 1000 epochs, reveals that NN5 consistently outperforms NN4. This enhancement can be ascribed, at least in part, to the employment of random transformations on the training image set, despite the fact that the evaluation image set was captured under identical lighting conditions and camera positioning as the training set.

The evaluation set, comprising 164 images, was segmented in 25.4 s, yielding a processing speed of 6.46 frames per second (FPS) or 0.15 s per image. Research indicates that, in comparison to other state-of-the-art networks, Mask R-CNN, a two-stage model, exhibits slower processing times [10,21]. However, given that the primary focus of this study was accurate instance segmentation rather than optimization for processing speed, the network’s processing time was deemed satisfactory based on the resulting precision of the models.

### 3.2. The Visual Comparison of Segmentation

In NN1 (Figure 8a), an excessive number of detections is observed, with seven detections instead of the expected four, which is unacceptable for practical use in an industrial environment. Furthermore, the detections themselves are not optimal, as the potato and the detector mask do not fully overlap. In NN2 (Figure 8b), it is evident that increasing the number of epochs not only improves the results but also enhances segmentation accuracy. The implementation of NN2 results in the detection of four potatoes, aligning with the actual number present in the image. Despite the appropriate placement of bounding boxes, errors can be observed in the detection masks, either encroaching upon the potatoes or extending beyond their boundaries. An intriguing wavy structure is evident in the masks of NN1, NN2, NN3, and NN4, all of which share the common parameter of being trained in the MATLAB environment. In NN3, a clear network error is evident, resulting in detections that are not suitable for industrial applications (Figure 8c). NN4 generates detections that are visually similar to those of NN2 yet are more precise in most cases (Figure 8d). However, an error is observed in the detection of the leftmost potato, where the detection mask exhibits an excess area on the lower side. Such an error could lead to the incorrect classification of potatoes in the sorting system. Among all segmentation results, NN5 produces the best detections, generating the most accurate masks that align perfectly with the actual potatoes, ultimately leading to the most precise segmentation results (Figure 8e).

## 4. Discussion

This study focused on training and applying a Mask R-CNN neural network to work in a potato sorter designed for industrial agriculture application. Within the study, the NN5 model demonstrated the most optimal outcomes, both visually and through computational evaluation. This outcome was influenced by several parameters, including the implementation of random alterations to training images and changing the number of epochs. The alternative software used with NN5 affected the segmentation speed of the model, thereby enhancing its usability in the production process, since the effectiveness of a neural network is determined not only by its accuracy but also by the speed of data generation. A notable characteristic of this neural network is its superior evaluation performance with a lower number of epochs, indicating that optimizing the learning parameters can improve the network’s overall performance. It was also observed that unpredictable errors might occur during the training of a neural network, potentially leading to malfunction and rendering the model unusable in a production environment. Therefore, the possibility of errors must be considered during training, which extends the learning process, as an additional model must be trained to ensure proper functionality. All of the wrong segmentation results in other models would be the result of training on too few epochs, since the segmentation results increased with the increase in the number of epochs.

The processing speed of a neural network directly impacts the efficiency of a sorting system. In this study, the trained models demonstrated a processing rate of 6.46 FPS. When utilized in conjunction with a wide-lens configuration, the sorting machine should exhibit the capacity to process multiple potatoes per second, with the precise number adjustable based on the available computational power. The utilization of advanced graphic cards is expected to yield enhanced performance, though it should be noted that this approach is accompanied by a higher cost for the sorting system. Testing on images that had not been previously encountered confirmed the efficacy of a region-based convolutional neural network in accurately segmenting potatoes within a realistic production environment. The subsequent stage involves integrating the neural network into an actual production process and evaluating the performance of the entire sorting system. While the focus would be integrating Mask R-CNN into a production environment, numerous industrial food-sorting systems rely on the use of classical computer-vision techniques (e.g., threshold-based segmentation and SVM-based classification). The good quality of such techniques is their ability to exhibit low computational demands, allowing for a higher quantity of produce being processed in equal amount of time. However, research has demonstrated that classical computer-vision techniques encounter challenges in complex segmentation tasks, particularly in scenarios involving occlusion, overlapping, and variable lighting conditions [28,29]. Conversely, deep learning models, such as Mask R-CNN, exhibit a marked advantage in addressing these issues by learning spatial relationships within the image and generating precise segmentation masks for each instance, albeit at the expense of a longer processing time. Leading industry entities such as TOMRA (TOMRA Systems ASA, Asker, Norway) and Key Technology (Key Technology, Walla Walla, USA) employ a suite of vision-based sorting techniques, including hyperspectral imaging and classical computer-vision algorithms, to automate food sorting at an industrial scale. While these systems are optimized for speed and efficiency, the focus of this work is on integrating deep learning-based segmentation methods, such as Mask R-CNN, which have the potential to enhance defect detection, produce classification, and object appearance handling.

It would be particularly interesting to observe how the neural network performs within a production sorter, where various parameters (e.g., changes in lighting conditions) may fluctuate. Such fluctuations could potentially lead to improper segmentation, which could result in a broken segmentation system. Following the observation, a subsequent evaluation of segmentation should be conducted, using images of potatoes on a working sorting system where the fluctuations would be clearly visible. Given that the sorting system utilizes a closed light box for the purpose of segmenting potatoes, the only variable that is expected to undergo change is the sharpness of the image. Upon implementing the sorting system, a cost calculation comparing the cost of manual labor to the cost of an automated sorting system would also be valuable. This should include factors such as the cost of electricity and the potential cost of a supervising worker. Another system enhancement could involve enabling the segmentation of different potato varieties, which could be achieved by expanding the dataset used for training the neural network. Future work should focus on optimizing inference speed, integrating real-time monitoring, and testing alternative deep learning architectures (e.g., YOLOv8, Swin Transformer) to further enhance segmentation efficiency based on speed and precision. The testing of the architectures could also include an analysis of the segmentation failures, encompassing false positives, false negatives, and occlusions. It would be interesting seeing where different models experience errors. The study could also continue with a focus on synthetic data augmentation to increase the training image dataset and evaluate the results of a neural network trained on such dataset.

## 5. Conclusions

This study explored the application of Mask R-CNN for segmenting potatoes in an industrial potato-sorting process, comparing different implementations and training variables to determine the most effective approach. The results highlight that while deep learning-based segmentation significantly outperforms traditional methods, the performance is highly dependent on software environment, training parameters, and data augmentation techniques. Among the tested models, the neural network (NN5) achieved the highest mean average precision (mAP = 0.878) and F1 score (0.597), demonstrating superior segmentation accuracy. The findings indicate that strategic selection of training variables and model optimization can yield substantial improvements in both accuracy and processing speed. Notably, increasing the number of training epochs does not always lead to better performance, emphasizing the importance of efficient model tuning and proper dataset preparation. Despite these promising results, several challenges remain for real-world deployment. Factors such as varying lighting conditions, potato shape irregularities, and high-speed conveyor operation must be addressed to ensure robustness in industrial applications.

Ultimately, this research confirms that Mask R-CNN is a viable solution for automated potato sorting, with potential scalability to broader agricultural applications. Implementing this approach in production environments could significantly improve sorting accuracy, reduce manual labor costs, and enhance overall efficiency in food processing industries.

## Figures and Tables

**Table 1:** Image distribution by category.

Classification	High-Quality	Feed	Rotten	∑	∑ [%]
Total images	216 31%	375 53.9%	105 15.1%	696	100
Training set	52 9.8%	375 70.5%	105 19.7%	532	76.4
Evaluation set	164 100%	0 0%	0 0%	164	23.6

**Table 2:** Parameter settings for function trainingOptions() in MATLAB.

Settings	Value
InitialLearnRate	0.001
LearnRateSchedule	»piecewise«
LearnRateDropPeriod	1
LearnRateDropFactor	0.95
Plot	»none«
Momentum	0.9
MiniBatchSize	2
BatchNormalizationStatistics	»moving«
ResetInputNormalization	false
ExecutionEnvironment	»gpu«
VerboseFrequency	50

**Table 3:** Learning parameters of neural networks.

Neural Network	Training Image Set Dimensions	Number of Epochs	Programing Environment
NN1	1920 × 1080	10	MATLAB
NN2	480 × 270	500	MATLAB
NN3	1280 × 720	500	MATLAB
NN4	480 × 270	1000	MATLAB
NN5	512 × 512	40	Python

**Table 4:** Evaluation results of neural networks.

Neural Network	Confidence Threshold	mAP	Average *F*1
NN1	0.7	0.562	0.486
NN2	0.7	0.675	0.494
NN3	0.66	0.005	0.032
NN4	0.7	0.741	0.526
NN5	0.52	0.878	0.597

## Data Availability

The original contributions presented in this study are included in the article. Further inquiries can be directed to the corresponding author(s).

## Abbreviations

The following abbreviations are used in this manuscript:

CNN	Convolutional neural network
R-CNN	Region-based convolutional neural network
P	Precision
AP	Average precision
mAP	Mean average precision
R	Recall
AR	Average recall
TP	True positive
FP	False positive
FN	False negative
IoU	Intersection over union
F1	F1 score
NNn	Neural network n
